# Deciphering the metabolic capabilities of Bifidobacteria using genome-scale metabolic models

**DOI:** 10.1038/s41598-019-54696-9

**Published:** 2019-12-03

**Authors:** N. T. Devika, Karthik Raman

**Affiliations:** 1 Department of Biotechnology, Bhupat Jyoti Mehta School of Biosciences, Indian Institute of Technology (IIT) Madras, Chennai, 600 036 India; 2 Initiative for Biological Systems Engineering (IBSE), IIT Madras, Chennai, India; 3 Robert Bosch Centre for Data Science and Artificial Intelligence (RBCDSAI), IIT Madras, Chennai, India

**Keywords:** Biochemical networks, Computational models, Biochemical networks, Computational models

## Abstract

Bifidobacteria, the initial colonisers of breastfed infant guts, are considered as the key commensals that promote a healthy gastrointestinal tract. However, little is known about the key metabolic differences between different strains of these bifidobacteria, and consequently, their suitability for their varied commercial applications. In this context, the present study applies a constraint-based modelling approach to differentiate between 36 important bifidobacterial strains, enhancing their genome-scale metabolic models obtained from the AGORA (Assembly of Gut Organisms through Reconstruction and Analysis) resource. By studying various growth and metabolic capabilities in these enhanced genome-scale models across 30 different nutrient environments, we classified the bifidobacteria into three specific groups. We also studied the ability of the different strains to produce short-chain fatty acids, finding that acetate production is niche- and strain-specific, unlike lactate. Further, we captured the role of critical enzymes from the bifid shunt pathway, which was found to be essential for a subset of bifidobacterial strains. Our findings underline the significance of analysing metabolic capabilities as a powerful approach to explore distinct properties of the gut microbiome. Overall, our study presents several insights into the nutritional lifestyles of bifidobacteria and could potentially be leveraged to design species/strain-specific probiotics or prebiotics.

## Introduction

The human gastrointestinal tract harbours a diverse and complex microbial ecosystem, which regulates multiple physiological processes that play a fundamental role in the well-being of their host. This microbiome is associated with a plethora of functions, which include fermentation and absorption of complex carbohydrates, maturation and normal development of immune functions, and prevents adhesion of pathogens to the intestinal surface^1^. Several factors, starting from the mode of delivery, to breastfeeding, gender, age, geography, disease, drug usage and long-term dietary intake, influence the structure and activity of the trillions of microorganisms inhabiting the gastrointestinal tract^2^. Many diseases, notably obesity, coronary heart disease, diabetes and inflammatory bowel disease, have all been associated with dysbiosis in gut microbiota composition^3^. Thus, the gut microbiome is considered a complex polygenic trait, shaped by both environmental and genetic factors^4^.

Bifidobacteria, most frequently isolated from the faeces of breast-fed infants, are involved in the maintenance of intestinal microbial balance and health. Bifidobacteria exert their biological activities through the production of vitamins and antimicrobial substances; further, they regulate the immune system and have anti-obesity and anti-inflammatory activities^5,6^. Bifidobacteria are gram-positive, anaerobic, and saccharolytic, and have been reported to inhabit the intestinal tract of mammals and insects, the human oral cavity and sewage^7^. This genus encompasses a broad range of enzyme-coding genes associated with the uptake and catabolism of complex and non-digestible carbohydrates, ranging from human milk oligosaccharides to plant fibre^8^. Bifidobacteria degrade the hexose sugars glucose and fructose through a unique pathway named “bifid shunt”, which is centred on the key enzyme fructose-6-phosphate phosphoketolase^9^. The metabolites from this ATP-generating pathway mainly produce short-chain fatty acids (SCFAs) that antagonise pathogenic bacteria and form a barrier against infection^10^. For instance, acetate produced by bifidobacteria improves intestinal defence mediated by epithelial cells and thereby protects the host against lethal infections^11^.

Several species of bifidobacteria such as *B*. *animalis*, *B*. *breve* and, *B*. *longum* are used to treat various gastrointestinal disorders and inflammatory bowel disease^12^. Also, a few strains of bifidobacteria such as *B*. *animalis* BF052, and *B*. *animalis subsp*. *lactis* BB-12 form the major functional ingredients in commercialised probiotic food products^13^. Notably, the probiotic characteristics of bifidobacteria are strongly strain-dependent^14^, with applications in the food, dairy, and pharmaceutical industries. Therefore, an understanding of the metabolism of this genus in its entirety and the adaptation of distinct strains to a variety of nutrient environments is definitely necessary.

In the recent decade, considerable effort has been invested into understanding the gut microbiome using metabolic modelling, particularly constraint-based reconstruction and analyses, which generate testable hypotheses to elucidate the metabolism of individual species, and also interspecies metabolic interactions^15–17^. Recently, Thiele and co-workers^18^ generated AGORA (Assembly of Gut Organisms through Reconstruction and Analysis), an excellent resource of semi-curated genome-scale models specifically for human gut microbes, enabling system-level studies of the gut. These genome-scale metabolic models of gut microbes have been used to predict growth phenotypes under different conditions and also provide a link between dietary intake and absorption in humans^19,20^. Moreover, research on strain-specific metabolic reconstruction has been explored for strains of *E*. *coli*^21^, *Staphylococcus aureus*^22^ and *Salmonella*^23^. These studies exemplify the use of metabolic networks to probe strain/species-specific diversity and provide insights into the utility of different strains towards their diverse applications.

The identification and classification of *Bifidobacterium* species have been demonstrated using DNA-DNA hybridisation^24^, and by creating a phylogeny based on whole and/or conserved genomic sequences^25^. In the present study, we set out to capture the diversity between strains of bifidobacteria by generating condition-specific metabolic models with the genome-scale metabolic models obtained from AGORA and subsequently investigating various phenotypic and metabolic characteristics. We simulated the models using two popular constraint-based analysis techniques—Flux Balance Analysis (FBA)^26,27^ and Flux Variability Analysis (FVA)^28^. We report pronounced differences across strains with respect to the nutrient utilisation, metabolic capabilities, variability in the bifid shunt pathway, and essential reactions under diverse niches. Ultimately, our modelling approach enabled us to classify the bifidobacteria into three groups, (i) *B*. *bifidum*, (ii) *B*. *animalis*, and (iii) *B*. *longum* based on multiple phenotypic and metabolic properties. In summary, this study employs a multi-pronged modelling approach to systematically characterise bifidobacteria based on their various phenotypic and metabolic features under different nutritional environments.

## Results

In this section, we describe our key results illustrating how our constraint-based modelling approach enables a careful classification of 36 strains of *Bifidobacterium* and contributes to a detailed understanding of their metabolism and metabolic capabilities.

### Bifidobacteria cluster into two groups based on carbohydrate utilisation

**Figure 1:**
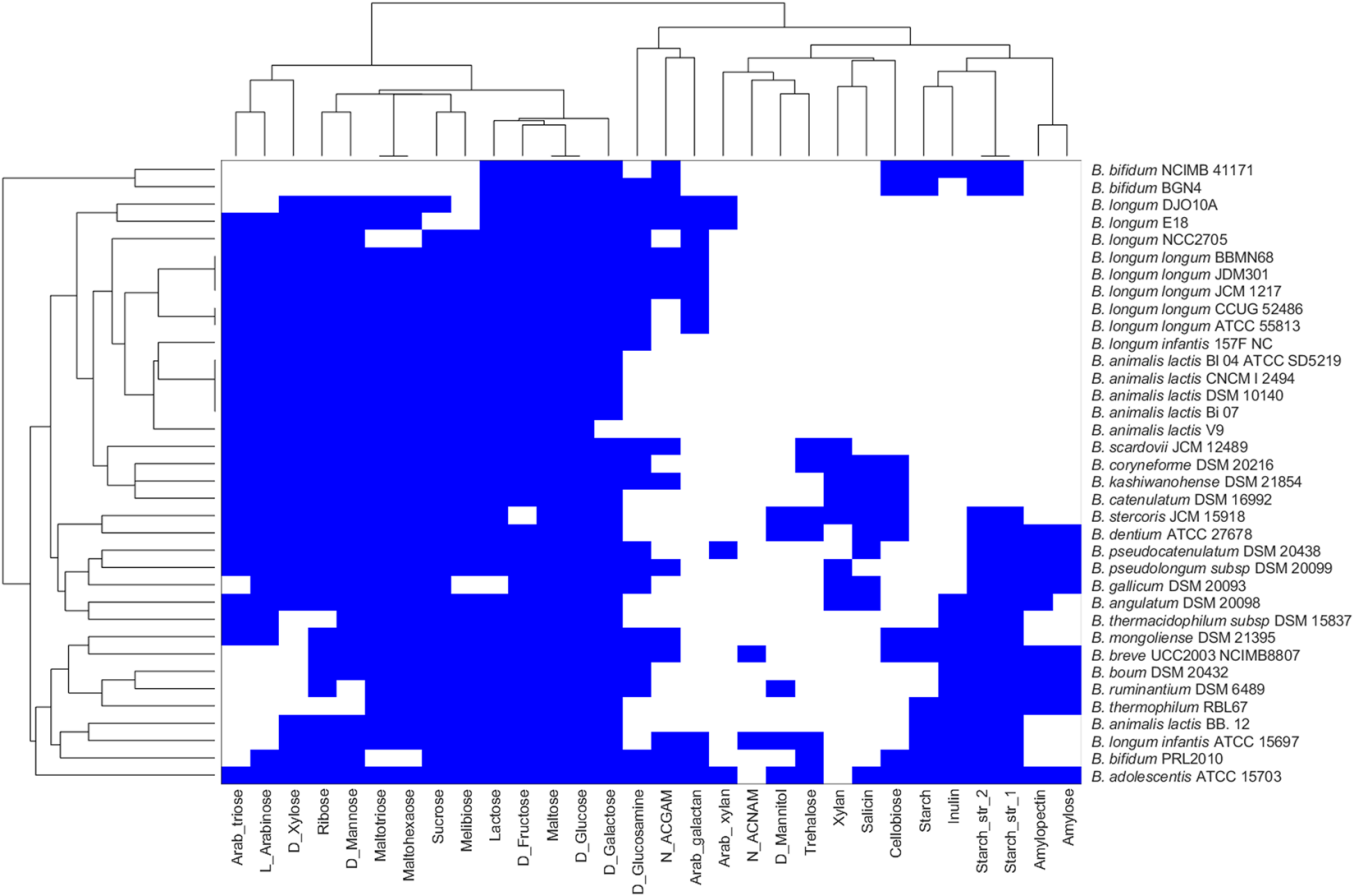
Clustering of bifidobacterial species grown on multiple nutrient environments. Rows represent individual strains and columns represent different nutrient environments. Strains are clustered based on the ability to sustain growth in each of the different nutrient environments. The presence and absence of growth are indicated by blue and white colours, respectively.

Based on the phenotypic prediction with respect to carbohydrate utilisation, the 36 strains could be differentiated into two groups, as shown in Fig. 1. All 36 strains included in this study showed *in silico* growth on glucose, fructose and maltose. The strains BGN4 and NCIMB 4117 demonstrated limited fermentation ability with predicted growth on 11 different carbon sources compared with other species of *Bifidobacterium*. Further, among the strains studied, *B*. *adolescentis* ATCC 15703 could utilise 28 of the 30 carbon sources used in this study, making the strain nutritionally versatile, followed by *B*. *dentium* ATCC 27678 and *B*. *kashiwanohense* DSM 21854 belonging to *B*. *adolescentis* group^25^. The probiotic strain *B*. *bifidum* BGN4 was the only strain that could not utilise the prebiotic carbohydrate inulin. In addition, we could observe that *B*. *longum infantis* ATCC 15697 differed from all the other strains from this same species in fermenting mannitol and trehalose and not fermenting arabinose and arabinotriose. All strains from the widely used commercial species, *B*. *animalis*, clustered together, with *B*. *animalis* BB 12 showing a distinct ability to ferment mannose. Overall, we observed distinct substrate utilisation profiles for our diverse collection of bifidobacterial strains.

### Lactate and acetate production vary across strains in different environments

To determine the metabolic capabilities of bifidobacteria, we performed Flux Variability Analysis, and the production of metabolites, namely acetate, lactate, ethanol, succinate, and formate, were assessed.

**Figure 2:**
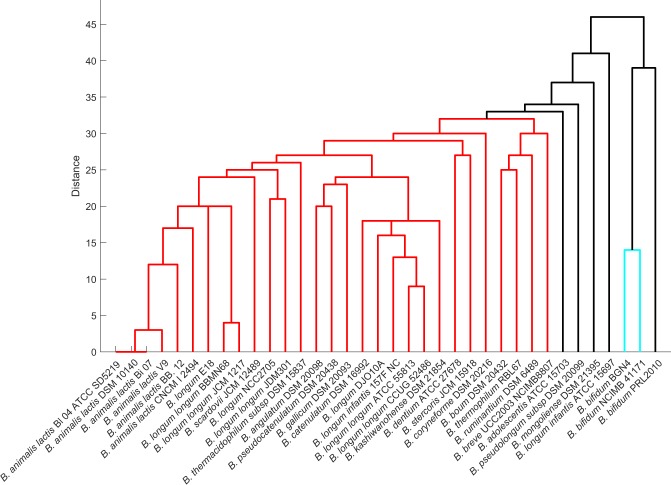
Dendrogram representation of bifidobacteria based on SCFA production. SCFA production of strains under diverse nutrient environments was represented as binary vectors (indicating presence or absence of SCFA production) and used to calculate the Hamming distance between strains.

Based on the clustering of the synthesis profiles of SCFAs in diverse nutrient environments, three major divisions can be distinguished among the bifidobacterial strains, as shown in Fig. 2. Among the SCFAs, the present study majorly focussed on acetate and lactate, which make a significant contribution to the prevention and treatment of metabolic syndromes^29^. Among the studied strains, *B*. *kashiwanohense* DSM21854, *B*. *gallicum* DSM 20093, and *B*. *longum infantis* 157 F NC were found to be producers of acetate as well as lactate in their survivable environments. The strain CNCM I 2494 could produce acetate in only three different environments, the least amongst the 36 strains considered. The strain *B*. *bifidum* PRL 2010 clustered with the nutritionally versatile strain *B*. *scardovii* JCM 12489. In contrast, *B*. *adolescentis* ATCC 15703, the strain which showed *in silico* growth on 28 different carbon sources could produce acetate in only 18 of those environments.

In contrast to acetate production, 29 out of 36 bifidobacterial strains could produce lactate across their survivable nutrient environments. The two strains of *B*. *longum*, NCC 2705 and JDM 301, lack lactate production when monosaccharides were used as the sole nutrient source, with a clear shift of flux towards formate production. Possibly, the assigned carbon uptake was found to be limiting for these strains of *B*. *longum*. Unexpectedly, the strains with lesser fermentation ability, i.e. the strains that showed *in silico* growth on only 11 carbon sources (Fig. 1), viz. *B*. *bifidum* BGN4, *B*. *bifidum* NCIMB 41171 and *B*. *bifidum* PRL2010, produced lactate in all (growth supporting) environments. Further, we could observe that polysaccharides, namely starch, inulin, maltohexaose, arabinotriose and arabinoxylan, displace the flux of *Bifidobacterium* strains towards lactate production. But with monosaccharides, a balance of acetate and lactate production is clearly evident (Supplementary Fig. 1).

We further explored the metabolic capabilities of bifidobacterial strains in a carbon-rich environment, by allowing uptake for all mono-, di-, tri-, and polysaccharides separately. As observed in the individual nutrient source utilisation, *B*. *animalis* CNCM I 2494 strain could produce acetate when allowed an uptake for all monosaccharides considered in the study and not with di-, tri-, or polysaccharides. In the case of *B*. *longum* strains, namely NCC 2705 and JDM 301, allowing uptake of all monosaccharides together resulted in lactate production. This observation concurs with the results mentioned above where the uptake of a single monosaccharide was found to limit lactate production. Taken together, our analyses show that the production of acetate turns out to be influenced by the nutrient environment and varies across strains. On the other hand, lactate production was independent of the nutrient environment.

### Dispensability of bifid shunt pathway in *Bifidobacterium* across nutrient environments

We simulated knock-outs of reactions associated with the bifid shunt pathway in order to understand how dispensable the reactions are, across bifidobacteria (Supplementary Table S5). We first categorised the reactions based on their ability to carry flux under different nutrient environments. Intriguingly, only seven reactions turned out to be carrying flux across all environments, and the remaining 19 reactions were found to be environment-specific. We designate such reactions as *conserved* and *non-conserved*, respectively.

**Figure 3:**
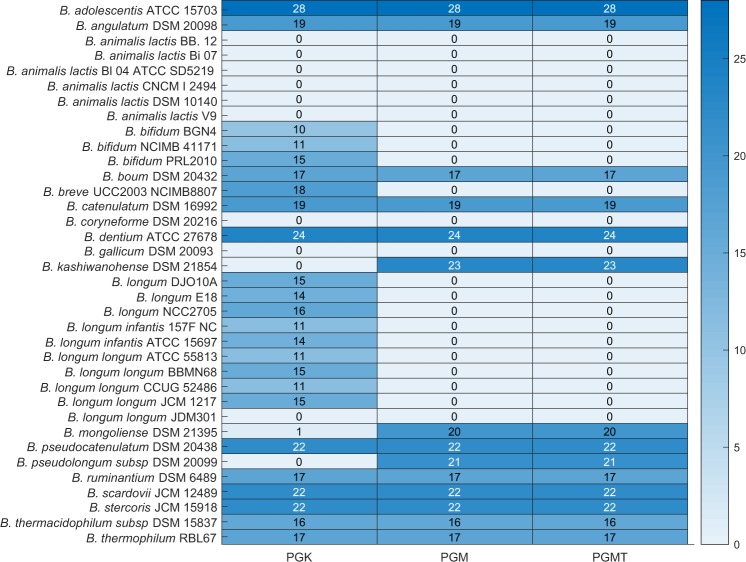
Overview of growth outcomes upon deletion of PGK, PGM, and PGMT in all 30 different nutrient environments. The rows represent 36 strains of bifidobacteria. The colour bar indicates the number of environments in which the organisms can survive upon deletion of PGK, PGM and PGMT individually.

We next performed a reaction knockout on the seven conserved reactions one at a time in each of the environments. We identified three reactions, namely phosphoglucomutase (PGMT), phosphoglycerate mutase (PGM), and phosphoglycerate kinase (PGK) with a major impact, and ribulose 5-phosphate 3-epimerase (RPE) with a lesser impact on the viability of strains. Deletion of the reactions PGK, PGM and PGMT abolish viability in all strains of *B*. *animalis* and other strains, namely *B*. *coryneforme* DSM 20216, *B*. *longum longum* JDM 301, and *B*. *gallium* DSM 20093 across all nutrient environments (Fig. 3). Among the nine probiotic strains analysed, only *B*. *thermophilum* RBL 67 was viable upon all three reaction knockouts performed across nutrient environments.

Thus, PGK, PGM, and PGMT are essential for nine strains across environments. Further, in our analysis, we identified the reactions PGM and PGMT to be essential for all strains of *B*. *longum* and *B*. *bifidum*. Taken together, we identified a set of species-specific environment-independent essential reactions. Further, the deletion of these enzymes contributed to the production of acetate in 10 different strains of bifidobacteria, in all their surviving environments. This is expected because deletion of either of these enzymes lowers the NADH availability, which in turn shift the flux towards acetate production.

In contrast, we found that 11 strains of bifidobacteria bypass these reactions and can sustain growth across environments. Therefore, we investigated the corresponding reaction pairs that maintain viability for these 11 strains. Here, we applied the concept of synthetic lethality to identify the reaction pair that rescued growth in many strains. Synthetic lethality analysis was performed using the Fast-SL algorithm, identifying single and double reaction lethals. Interestingly, in the 11 strains of bifidobacteria which bypass these reactions, these enzymes (PGK, PGM and PGMT) were found to be double lethal.

### Reaction essentialities in a rich environment reveal diversity between strains

**Figure 4:**
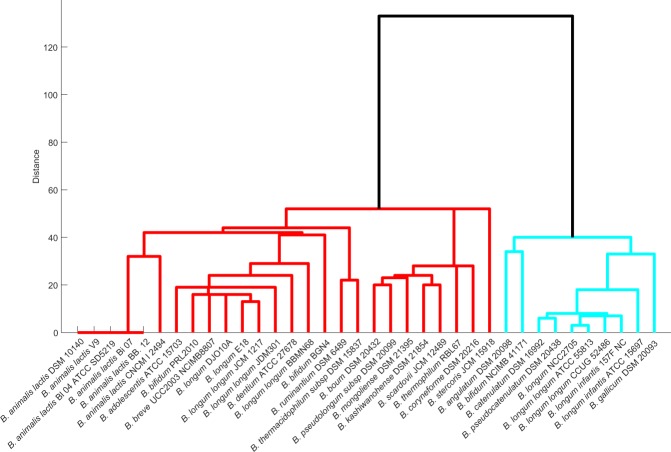
Dendrogram representation of Bifidobacterial species based on the presence of single lethals. Organisms were grouped based on the similarity of their single lethals, generated using Fast-SL on the rich environment. Each organism’s single lethal profile was represented as a binary vector (see text) and used to compute the similarity between strains.

We here identify reactions essential for the survival of the genus *Bifidobacterium* using the Fast-SL algorithm in a rich environment and analyse to what extent this essentiality is conserved across species/strain. Among the set of organisms used in the study, *B*. *ruminantium* DSM 6489 and *B*. *gallicum* DSM 20093 carry the maximum and the minimum number of essential reactions, respectively (Supplementary Table S8). We find 459 reactions (excluding exchange reactions) to be essential across strains of bifidobacteria. Out of these 459 reactions, 169 were found to be the *core set* of essential reactions, that is, they were present in all the 36 strains. Next, we investigated in which biological pathways these essential reactions function. As expected, most of the essential reactions significantly fall in pathways that synthesise cell wall components, amino acids metabolism (phenylalanine, valine, leucine and isoleucine) and purine synthesis. The grouping of the strains by the effect of essential or single lethal reactions revealed two major classifications of *Bifidobacterium*. The ten strains of bifidobacteria from Group 1 (Fig. 4) carry fewer single lethal reactions compared to the total essential reactions of other strains.

### Bifidobacteria classify into three distinct groups based on metabolic capabilities

Consolidating all the analyses reported above, we classify the bifidobacteria into three distinct groups: (**1**) The strains of the species, *B*. *bifidum*, namely NCIMB 41171 and BGN4 are well separated from all 34 strains of *Bifidobacterium* based on their phenotypic predictions, SCFA production, as well as local differences within the bifid shunt pathway. (**2**) The strains of *B*. *animalis*, used specifically in the food industry, represent a group with highly similar phenotypic and metabolic characteristics. In particular, they form a cluster with *B*. *longum*, based on growth and with local differences in the bifid shunt pathway. On the other hand, it forms a cluster with *B*. *thermacidophilum subsp*. *thermacidophilum* DSM 15837 based on the SCFA production. (**3**) The ten strains of *B*. *longum*, being from the same species, however, are not identified as one group due to diverse nutrient and metabolic capabilities. The strains *B*. *longum longum* ATCC 55813, *B*. *longum longum* CCUG 52486, and *B*. *longum infantis* 157 F NC tend to group based on phenotypic and metabolic characteristics. These three groups distinguish themselves based on their metabolic properties and naturally lend themselves to different applications.

## Discussion

Bifidobacteria are the quintessential gut inhabitants, which modulate the metabolic and immune activities of the host^30^. Their ability to utilise a wide variety of complex substrates reflects their metabolic adaptations^31^. It is well-known that even closely related strains vary in their metabolic repertoire^32^. Therefore, in the present study, emphasis has been laid on genome-scale metabolic networks, as they elucidate the differences with respect to the metabolic capabilities of the organisms. The genome-scale metabolic networks used in this study^18^ represent the entire metabolic repertoire of *Bifidobacterium*. These metabolic models were further curated, to enable the simulation of condition-specific models with a defined set of universal media components.

We began our analysis by computing growth phenotypes on the curated models in 30 different nutrient environments to determine the distinction between strains. The higher fermentation ability exhibited by *B*. *adolescentis* in this study possibly highlights the metabolic gains made by these species upon adapting to human colon^33^. The limited fermentation ability observed in *B*. *bifidum* strains correlates with a relatively lower number of carbohydrate transport systems compared to other intestinal bifidobacteria^34^. Remarkably, throughout our analysis, all investigated *B*. *animalis* strains were clustered together indicating homogeneity and a high degree of genome conservation in these strains as reported^35^. Moreover, the ability of bifidobacterial strains to ferment common saccharides, including polysaccharides such as amylopectin, pullulan, maltotriose, and maltohexaose, render them competitive in the gut environment. We also noticed that an effective prebiotic inulin which promotes the proliferation of bifidobacteria^36^, is utilised by the adult as well as the infant strains of bifidobacteria excluding any relationship between strain origin and fermentation ability. These analyses reveal the extensive fermentation capabilities of distinct strains of bifidobacteria.

Next, we explored the secretion of SCFAs, acetic and lactic acid, as it protects the host against lethal infection^37^. The capability of the probiotic candidate, *B*. *thermophilum* RBL67 to produce acetate and lactate observed in our study could be one of the contributing factors for its antagonistic and protective effects against *Salmonella* and *Listeria* species^38,39^. The inability of *B*. *longum* strains, NCC2705 and JDM301, to produce lactate with monosaccharides and enabling flux to formate, is also reported in the strain *B*. *longum subsp*. *longum* UCD401, which secrete high concentrations of formate with xyloglucans as substrate^40^. It must be pointed out that bifidobacterial secretion of formate while utilising glucose has also been previously observed^41^. Studies of Wang *et al*.^42^ show the importance of *Bifidobacterium* in relieving constipation mainly by improving the concentration of acetic acid in the intestine. Our study identified *B*. *gallicum* and *B*. *kashiwanohense* to be capable of producing acetate in all viable environments. We hypothesise that these strains would also contribute to maintaining acetic acid levels in the intestine. Overall, the contribution of acetate/lactate production by each strain was found to be variable across nutrient environments, revealing the need to understand the strain-specific differences that could probably give a clue for the selection of the most relevant strains for distinct applications.

We further considered the bifid shunt pathway and studied how variable or conserved are the reactions in the pathway across strains under multiple nutrient environments. We found that the enzymes PGK, PGM and PGMT from the bifid shunt pathway exhibited a major impact on the viability of a subset of strains across all nutrient environments. However, given the fact that all strains share the enzymes, PGM and PGK were essential to only a subset of species/strains. This highlights the existence of a unique network structure, where strains take up alternative metabolic routes or compensatory reactions to furnish energy and biomass precursors necessary for growth. The reaction essentialities enabled us to identify two main clusters of *Bifidobacterium*, reflecting species/strain-specific differences in the metabolic reactions. We identified a core set of 169 reactions, essential to all bifidobacteria—given the central importance of bifidobacteria in human health, it will be important to make sure that new antibiotics do not interfere with this core set of essential reactions.

This work highlights the potential of constraint-based approaches, to understand the contribution of individual species/strains towards fermentation and production of metabolites that will help in identifying the relevant strains with diverse applications. Our study does have its limitations. Firstly, we are limited by the number and quality of the genome-scale reconstructions presently available^43^. Consequently, some of the strains in this study are not as well-represented as others; while we have six strains of *B*. *animalis*, we have only one strain *of B*. *adolescentis* ATCC 15703. Further, BLASTP homology searches performed do not necessarily ensure that the corresponding enzymes are functional, which needs further experimental validation. As no experimental constraints (e.g. internal flux measurements arising out of ^13^C metabolic flux analysis) were used in the model, it should be noted that the variations observed between distinct strains must be taken in a more qualitative context, rather than quantitatively, in terms of the exact values of the fluxes. Finally, our studies unravel three distinct clusters of these 36 strains, based on multiple phenotypic and metabolic properties. Together, these approaches provide a firm basis to understand the metabolic diversity of bifidobacteria and their roles in maintaining gut health.

## Conclusion

Overall, the present study lays special emphasis on the nutrient utilisation, metabolic capabilities, and the bifid shunt pathway of *Bifidobacterium* species. With the refined genome-scale metabolic models of bifidobacteria, we anticipate that regardless of whether from the same species, we observed inter-and-intra-strain differences at fermentation and metabolic capability. Taken together, the analyses mentioned above present a comprehensive summary of knowledge regarding the metabolism of *Bifidobacterium*. We believe that the deeper understanding of bifidobacterial metabolism thus obtained will better facilitate the use and exploitation of bifidobacteria in various commercial applications.

## Methods

### Data

The genome-scale metabolic models of *Bifidobacterium* were obtained from Virtual Metabolic Human (VMH) Database^44^, a resource of semi-curated models of gut microbes, AGORA. AGORA contains 39 strains of *Bifidobacterium*, of which we chose 36 strains for our analysis. We eliminated three other strains (*B*. *animalis lactis* AD011, *B*. *bifidum* S17, and *B*. *breve* HPH0326), as they produced growth even in the absence of the representative carbon source glucose. The 36 strains (covers 20 different species) investigated in this study and the general information on the origin of these species are listed in Supplementary Table S1. Among these twenty species, *B*. *breve*, *B*. *bifidum*, and *B*. *longum* are the most prominent species in the infant gut^45^. Several of the other species used in this study are even commercially important, often used as a probiotic supplement.

### Model expansion to account for experimental growth profiles

We simulated a defined set of media components reported in the literature to achieve *in silico* growth for validation of the model^46^. We manually re-curated 36 genome-scale models of bifidobacteria to improve their fit to experimental observations in utilising the different carbon sources (Supplementary Table S3). During our analyses, most of the models failed to show growth on carbon sources where growth has been demonstrated in previous experiments. For example, except *B*. *adolescentis* ATCC 15703, none of the AGORA models demonstrated growth in the presence of starch. However, it is well-documented that most strains of bifidobacteria do hydrolyse starch^47^. The AGORA models failed to capture such starch utilisation, as most of the models lack reactions pertaining to starch metabolism. Therefore, we carried out a standalone BLASTP (with an E-value cut-off of 10^−5^) to identify putative enzymes associated with starch degradation across different strains. We observe that 31 strains of bifidobacteria do carry a homologue of the enzymes oligo-1, 6-glucosidase, *α*-1,6-pullulanase and *α*-1,4-amylase associated with starch degradation. Therefore, we added the associated reactions to the models corresponding to these 31 organisms. The remaining strains, namely *B*. *boum* DSM 20432, *B*. *coryneforme* DSM 20216, *B*. *thermacidophilum subsp*. *thermacidophilum* DSM 15837, *B*. *pseudolongum subsp*. *pseudolongum* DSM 20099 and *B*. *ruminantium* DSM 6489 lack the corresponding enzymes. The exact modifications like reaction addition performed to each of the models are listed in Supplementary Table S7.

### Defining universal media for bifidobacterial growth

A common universal *in silico* media was defined by removing the unlikely growth requirement components across the strains. The components identified as essential by at least one of the strains were added to the universal growth media. Finally, we generated an augmented set of universal components (Supplementary Table S2)., which can produce all biomass components across strains with glucose as the representative carbohydrate source.

### Growth simulation using flux balance Analysis (FBA)

The *in silico* growth prediction (i.e. the presence or absence of growth) for each of the 36 metabolic networks of bifidobacteria was performed using FBA. FBA is a reliable method to predict the metabolic capabilities of an organism, by estimating the fluxes of reactions in a metabolic network^48^. FBA uses a linear programming formulation to calculate the flux distributions, with the assumption that the system is in steady-state^49^. We used 30 different carbon sources/nutrient environments encompassing mono-, di-, tri-, and polysaccharides (Supplementary Table S3). The model growth phenotype was determined by allowing the uptake of only one carbon source at a time. In each of the conditions, we employed maximisation of biomass as the objective function. For the simulation of growth on different carbon sources, the lower bound of the corresponding carbon uptake was set as −10 mmol/gDW/h (Supplementary Table S3). For example, if glucose is used as the single carbon source, the lower bound of this corresponding exchange reaction was set to −10 mmol/gDW/h, with the lower bound of all other carbon sources set to 0. The universal *in silico* media was simulated by setting the lower bound to −1 mmol/gDW/h^29^ for each of the components.

### Metabolite production using flux variability analysis (FVA)

Each of the 36 models was optimised for metabolite production (acetate, lactate, ethanol, formate, and succinate). FVA calculates the flux range of each reaction by maximising or minimising the flux through the reactions^28^. For metabolite production, the biomass reaction was constrained to be equal to the maximum growth rate achieved. The models with flux value above 0.01 mmol/gDW/h were considered as metabolite-producers, whereas models with flux value below 0.01 mmol/gDW/h were considered as metabolite-non-producers.

### Reaction knockouts

For analysing the essentiality of bifid shunt pathway across *Bifidobacterium*, we first performed FVA on the 26 reactions (Supplementary Table S5) from the bifid shunt pathway in 30 different nutrient environments. Subsequently, we categorised the reactions as conserved and non-conserved, based on their ability to carry flux in the 30 environments. Further, we performed *in silico* single reaction knockouts in these reactions in all nutrient environments and computed the presence/absence of growth as well as the acetate/lactate production across environments. The reaction knockout was carried out by setting the bounds (lower and upper bounds) of the corresponding reaction(s) to zero.

### Identification of synthetic lethals using Fast-SL

We identified single and double lethals across each of the strains of bifidobacteria, using the Fast-SL algorithm previously developed in our laboratory^50,51^. The Fast-SL algorithm efficiently identifies lethal reaction sets by reducing the search space, under different growth conditions. The double lethals are pairs of reactions, where only the removal of both reactions abolishes the growth of the organism—removal of either of the reactions in the pair alone does not affect growth. The single and double lethal pairs were generated on rich media environment (Supplementary Tables S8 and S9). The rich environment was simulated by providing all the 30 carbon sources along with the common universal media.

### Clustering the bifidobacterial strains

For each organism, we simulated the presence/absence of growth in each of the environments (Fig. 1) and represented these observations as a binary vector (0 representing no growth, and 1 representing growth). We hierarchically clustered the organisms, using the average linkage algorithm as implemented in MATLAB clustergram, based on the Hamming distance between these binary vectors. This distance essentially captures how similar the growth *profile* of a pair of organisms is. Similarly, we generated binary vectors from the SCFA simulations, capturing the presence or absence of production, for each strain in each environment (Fig. 2). These vectors were used to compute the Hamming distances and cluster the organisms. We also generated the single lethals for each of the strains on rich environment using Fast-SL and represented the lethality profile of each organism using binary vectors. The binary digits in these vectors indicate whether or not each reaction is lethal in the organism. We then computed the Hamming distance between all pairs of organisms, to cluster the strains (Fig. 4). We generated clustergram and dendrogram plots using the MATLAB functions for illustrating the clustering obtained. A heatmap (Fig. 3) was generated to display the ability of the strains to grow on different carbon sources upon blocking the enzymes PGMT, PGM, and PGK.

### Model simulations

All simulations were performed on MATLAB 2016a (Mathworks Inc., USA) using the COBRA Toolbox version 2.0^52^ and *Gurobi6* (Gurobi Optimization LLC, USA) as the solver for solving the optimisation problems corresponding to FBA and FVA. All the expanded 36 genome-scale models and codes used for performing the simulations are available on GitHub (https://github.com/RamanLab/Curated-Bifidobacterial-GSM).

## Supplementary information

Supplementary Fig 1

Supplementary Tables

## Data Availability

All data generated and analysed in this study are available in the supplementary information files and on the companion GitHub repository.
