# Supplementary material for: Deciphering the metabolic capabilities of Bifidobacteria using genome-scale metabolic models
Source: Sci Rep. 2019 Dec 3;9:18222. doi: 10.1038/s41598-019-54696-9 (PMC6890778; doi:10.1038/s41598-019-54696-9)
Supplement: Supplementary file 1 — Supplementary Fig 1 [file 41598_2019_54696_MOESM1_ESM.docx]

Deciphering the metabolic capabilities of Bifidobacteria using genome-scale metabolic models

N. T. Devika^1, 2^ and Karthik Raman^1, 2, 3*^

*^1^Department of Biotechnology, Bhupat Jyoti Mehta School of Biosciences, Indian Institute of Technology (IIT) Madras, Chennai – 600 036, India*

*^2^Initiative for Biological Systems Engineering (IBSE), IIT Madras*

*^3^Robert Bosch Centre for Data Science and Artificial Intelligence (RBCDSAI), IIT Madras*

#
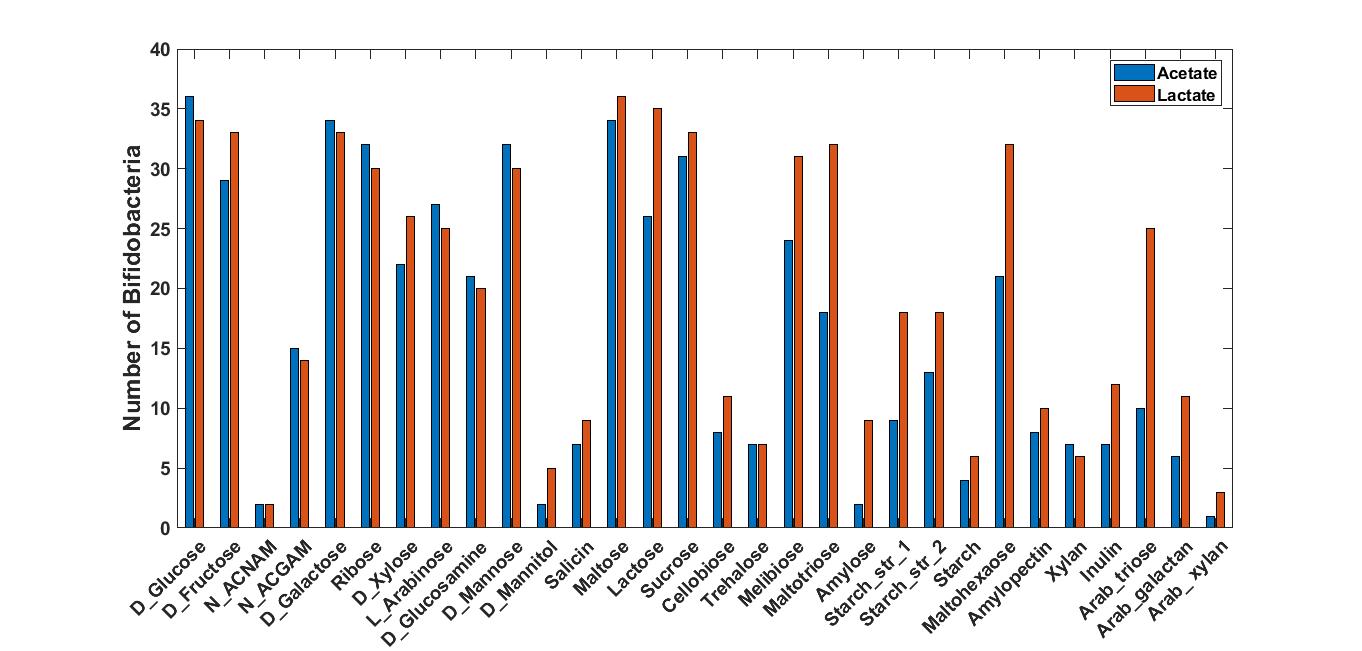
Supplementary Information

**Supplementary Fig 1:** **Environment dependent production of acetate and lactate across strains of bifidobacteria.**  Comparison of acetate and lactate production across strains of bifidobacteria under 30 different nutrient environments.
